# Conspecific chemical cues facilitate mate trailing by invasive Argentine black and white tegus

**DOI:** 10.1371/journal.pone.0236660

**Published:** 2020-08-12

**Authors:** Shannon A. Richard, Isabella M. G. Bukovich, Eric A. Tillman, Sanjiv Jayamohan, John S. Humphrey, Paige E. Carrington, William E. Bruce, Bryan M. Kluever, Michael L. Avery, M. Rockwell Parker

**Affiliations:** 1 Department of Biology, James Madison University, Harrisonburg, VA, United States of America; 2 National Wildlife Research Center, Florida Field Station, U.S. Department of Agriculture, Gainesville, FL, United States of America; Claremont Colleges, UNITED STATES

## Abstract

Squamate reptiles (snakes and lizards) rely on chemical cues from conspecifics to search the environment for potential mates. How such cues are used by invasive species to facilitate reproduction, especially seasonally, is a key question that can inform management practices. The Argentine black and white tegu (*Salvator merianae*) is an invasive reptile species in south Florida threatening native fauna in biodiverse regions such as Everglades National Park. While some information exists on the reproductive ecology of this species in its native range in South America, the chemical ecology of *S*. *merianae* is unclear especially in its invasive range. By testing both male (*n* = 7) and female (*n* = 7) tegus in a Y-maze apparatus, we assessed if either sex follows chemical trails left by conspecifics and if behaviors were sex- or season-specific. We conducted three types of trials where conspecifics created odor trails: Male-only (male scent only in base and one arm of Y), Female-only, and Male vs. female. Males did not preferentially follow scent trails from either sex, but they did differentially investigate conspecific scent from both sexes. Seasonally, males showed increased rates of chemosensory sampling (rates of tongue-flicking) during the spring (breeding season; March-May) compared to fall (non-breeding season; September-November). Males also had reduced turning and pausing behavior while trailing in the spring. Female tegus exhibited stronger conspecific trailing abilities than males, following both male and female scent trails, and they explored the maze less before making an arm choice. Females also investigated the scent trails intensely compared to males (more passes in scented arms, more time with scent trails). Our results demonstrate for the first time that females of an invasive reptile species can follow conspecific scent trails. Given the strong female responses to odor, sex-specific targeting of tegus via application of a conspecific chemical cue in traps could enhance removal rates of females during the breeding season.

## Introduction

Selection of potential mates is a crucial choice that typically determines offspring quality and survival. Mates are therefore heavily scrutinized and selected according to the quality of multiple sexual signals. Squamate reptiles (snakes and lizards) use chemical signals in mate choice to assess broad characteristics such as sex, mating history, body condition, and population [1]. For example, male red-sided garter snakes (*Thamnophis sirtalis parietalis*) evaluate a female’s sex pheromone composition to determine her size and condition—characteristics that directly affect female reproductive output [2, 3]. Likewise, male lizards of many species can determine sex, condition, and species using chemical cues, enabling distinction of mate qualities such as gravidity [4, 5]. Sexual chemical discrimination is enabled by specific compounds—pheromones—that can be produced by the skin or from specialized glands [6–8]. In many lizard species, sex pheromones enable female mate choice, where multiple male qualities can be assessed via these territorial scent marks [9]. Accordingly, females prefer scent marks from males in better condition (i.e., more symmetric, better immune response) indicated by increased rates of tongue-flicking or greater time allocation in conspecific home ranges [10, 11]. While scent marking by male lizards is the primary mode for sexual chemical signaling, males of several species also follow female scent trails [12–14].

Individual behavioral responses to integumental and cloacal chemical cues are commonly quantified in studies on squamate reproduction to interpret how receivers are interpreting the composition of said cues from conspecifics [6, 15, 16]. The most common behavior quantified in studies on squamate reptiles is tongue-flicking, which is a reliable indicator of an individual’s interest in a single cue or mixture of cues [17, 18]. Males in many lizard species exhibit a higher rate of tongue-flicking to female scent compared to male scent, and this response is often seasonal [4, 5, 19, 20]. A key example comes from jewel lizards (*Liolaemus tenuis*) where both sexes exhibit increased interest in female scent during the breeding season but not after [21]. Chemical cues are thoroughly studied in lizards for their roles in intrasexual aggression via male territoriality and mate competition, where substrate licking and rubbing are commonly exhibited behaviors [6]. In multiple species, male responses are contextual: they respond with courtship behavior to female scent and with aggression to male scent [22–25].

Chemosensory behaviors can indicate if focal animals are discriminating between conspecific scents and thus may provide promising utility in invasive species management decisions. Conspecific chemical cues used in mate location have significant potential for implementation in trapping given their historic utility in invertebrate and vertebrate pest management [26–28]. Our study is focused on an invasive reptile, the Argentine black and white tegu (*Salvator merianae*). Argentine tegus are terrestrial lizards with a native range widely distributed in South America East of the Andes that have successfully invaded the islands of San Andres, Colombia [29], and Fernando de Noronha, Brazil [30], as well as portions of southern Florida. As of August, 2019, > 4,700 observations of *S*. *merianae* were reported in the wild in Florida [31]. In 2019, an instance of early detection for *S*. *merianae* occurred in Toombs and Tattnail counties, Georgia, USA, approximately 600 km north of established populations in Florida [32]. This particular invasive reptile has the potential for significant, rapid rates of expansion [33–34]

Although the reproductive ecology of Argentine tegus is well understood, information on their chemical communication is lacking. In their native and invasive ranges, tegus brumate in burrows during the winter [35–38]. Mating season occurs during the spring after brumation when the ovaries are still previtellogenic [39]. Seasonal changes in circulating testosterone and estradiol levels show maxima during the spring (September-December; South America) mating season [40–42]. The increase in testosterone in male Argentine tegus is correlated with mating behaviors and increases in femoral gland secretions, which are commonly used in lizards in scent-marking behaviors (rubbing thighs on the ground) to delineate territories [39–40, 43–47]. Biochemically, the composition of the femoral secretions in male tegus changes during the mating season, and similar compositional changes have been recorded for green iguanas (*Iguana iguana*) [48,49]. Though intrasexual male-male signaling via chemical cues is presumed, there is anecdotal evidence that males also identify females via conspecific chemical cues. Male *S*. *merianae* have been documented courting a dead female, indicating that chemical cues may be sufficient to drive behavior [50]. A more comprehensive understanding of tegu conspecific chemical discrimination is warranted to understand the role and utility of such cues in managing this invasive species.

Invasive Argentine tegus pose a significant conservation and management concern for several reasons. Their burrowing behavior and high tolerance to cold temperatures facilitate the species’ potential to invade much of the southeastern United States [33]. *S*. *merianae* are also predacious omnivores, and established invasive populations threaten native wildlife, especially birds and reptiles which occupy burrows or nest on the ground [51–53]. In addition, the species exhibits relatively rapid maturation, high reproductive output, large body size, and a relatively long lifespan [54]. There is evidence to suggest that *S*. *merianae* will pursue and kill, but not consume native reptiles [55]. This combination of competitive and predatory behavior, known as intraguild predation [56], may exacerbate the impact of *S*. *merianae* on native reptile populations. Further, due to their omnivorous diet, *S*. *merianae* represent potential threats to other resources, particularly economic losses to agricultural industries [57]. Despite intensive efforts to eradicate established invader populations of *S*. *merianae*, there are no known instances of extirpation by way of hunting/culling [33]. This suggests current management tools available to managers, predominantly various configurations of live traps baited with eggs [58], are inadequate, and additional methods are needed to protect native wildlife communities from invasive tegus.

Our study is the first to assess trailing behavior in *S*. *merianae*. We tested the chemical trailing behavior of male and female Argentine black and white tegus in a Y-maze apparatus. Primarily, we sought to determine if tegus discriminate between sexes of conspecific chemical trails, if there are any sex-specific patterns in behavior that occur in the presence of varying chemical cues, and whether such behavior is seasonal. Our results will inform future directions for applied research and wildlife management strategies focused on this invasive reptile.

## Materials and methods

### Study species and husbandry

Male (*n* = 7) and female (*n* = 7) Argentine black and white tegus (*Salvator merianae*) were caught in the vicinity of Homestead, FL (Miami-Dade County) and brought to the U.S. Department of Agriculture’s National Wildlife Research Center (NWRC) field station in Gainesville, Florida, USA. Tegus were housed individually in outdoor pens (3.1 x 1.5 x 1.8 m; L x W X H) made of plastic coated wire on aluminum frame. Each pen had a burrow shelter which consisted of a polyethylene box (64 x 48 x 30 cm; L x W x H) with insulated top to which a 1-m length of 15-cm diameter black corrugated drain pipe was affixed. The burrow shelter and pipe were completely covered with up to 61 cm of dirt, with the exception of the insulated lid of the shelter and the distal end of the pipe which was left open for entry. The burrow shelters were used by the animals for daily thermoregulation, nighttime shelter, and as hibernacula during times of seasonal dormancy and brumation. Food rations were offered one to three days each week, depending on animal activity and appetite; water was offered *ad libitum*. Tegus were captured and brought to the field station at different times (2010, 2013, 2018), but all animals were acclimated to captivity and spent at least one winter in the outdoor pens prior to testing. Spring trials were timed to start with the onset of breeding activity which was defined as the time after brumation when we observed a marked increase in basking and pacing activity by both males and females. Trials for males were conducted from 9 April 2018 to 11 May 2018 and for females from 20 April 2019 to 24 May 2019. This timing is later than the identified breeding season for tegus in Florida (March) [59], but the delay is expected given the differences in the climate of south Florida, where most research to date has been conducted, to north Florida where our captive animal research took place. At 4 degrees higher in latitude, average temperatures in north Florida in winter and early spring are colder and cold temperatures persist longer, delaying the onset of breeding activity. Fall trials were conducted with only males from 1 September 2017 to 7 November 2017. All methods involving the use of live vertebrates were approved by the IACUC of the U.S. Department of Agriculture (study protocol QA-2901), and collection and housing of wild vertebrates was approved by Florida Fish and Wildlife Conservation Commission.

### Experimental apparatus

A Y-maze was used to run all scent trail tests and has been described elsewhere [60]. The Y-maze had an initial 1.4 x 0.42 m (L x W) passageway leading from the start box (1.08 x 0.56 x 0.46 m; L x W x H), ending in a 45° Y-junction from which two 1.2 x 0.40 m (L x W) passageways proceed to collection boxes (0.83 x 0.5 x 0.44 m; L x W x H). The arms of the maze were made of 2.5 x 15.2 cm (W x H) PVC side boards over which were attached a clear acrylic top to allow camera observations throughout the trials (S1 Fig). After assembly, the maze was attached to a 2.4 x 2.4 m “Hardie board” base. The start box and collection boxes (modified plastic storage bins) had reinforced door openings which attached to the arms of the maze. Removable acrylic doors allowed release and capture of animals in the Y-maze environment. The maze was secured within a locked outdoor pen (6.1 x 3 x 1.8 m; L x W x H). The pen was situated under a canopy to shelter the maze and surveillance cameras from rain. The top and sides of the pen were equipped with shade cloth to provide relief from the sun. For each trial, the floor of the maze was covered with plastic sheeting and then white Kraft paper, which provided a scenting surface for each trial. Trials were conducted between 1000 and 1700 hours. All pieces of the maze (top, sides, and holding boxes) were thoroughly cleaned with Micro® laboratory cleaner and water and air-dried between trials. The base was covered with new plastic sheeting and Kraft paper before starting the next trial. Disposable nitrile gloves were worn when washing and assembling the apparatus and boxes.

### Trials

Before testing experimental scent trails, bias tests were conducted where no scent was present in the maze. There was no bias in arm choice for either sex in the Y-maze (males: 2/7 chose the North arm, P = 0.224; females: 3/7 chose the North arm, P = 0.5).

For each set of experimental trials, tegus were assigned to trial types in a fully randomized design. Each trial allowed a focal tegu to explore a maze that presented one of three types of scent trail scenarios: Male-only, Female-only, and Male vs. female. Males and females experienced all trial types in Spring. In Fall, only males were run through two trial types (Male-only; Female-only). To create a scent trail, a randomly chosen stimulus animal was selected, placed in the holding box affixed to the base of the Y-maze, and allowed to acclimate for approximately 60 min. The door to the holding box was then opened, and the stimulus animal was allowed to move through the base and only one arm (the other arm was blocked with a partition). The open arm was randomly selected. Upon moving into the holding box at the end of the arm, the stimulus tegu was then removed as was the partition. In the Male vs. female trials, the base arm was divided lengthwise by a middle partition such that the stimulus animal could only scent the left or right half of the base paper before moving into the open arm. Then, the base arm partition was flipped and the second stimulus animal was run through. When the focal tegus were male in Male vs. female trials, male scent was laid first, then female. The opposite was the case with female focal tegus. Once the scent trail was created, the focal tegu was then placed in the holding box at the base of the maze, acclimated for approximately 60 min, then allowed to explore the maze. The trial was considered completed when the focal tegu’s head crossed into the holding box at the end of the chosen arm.

### Response variables and behaviors

Each trial was recorded using three digital surveillance cameras: one facing the start box to view the base arm, and one positioned above each collection box to view the arms of the Y. Videos were recorded directly onto a network video recorder, and videos were analyzed at James Madison University. Arm choice, choice penalty score, rate of tongue-flicking (tongue-flicks per min), pauses, turns, passes through each arm, and various trailing times were recorded. A tegu was considered to have made a choice once its head entered the holding box at the end of an arm; however, behaviors were also continually scored throughout the entire video. Choice penalty has been used in other studies of chemical trailing in reptiles [e.g., 60,61]. To assign a choice penalty score, both arms of the maze were divided into five 30 cm segments, and each tegu was given a negative point for each segment they moved into in the non-target arm (blank in the Male-only and Female-only trials; male in Male vs. female trials). The more negative the score, the farther into the non-target arm the tegu moved before choice occurred. Rate of tongue-flicking was recorded as the number of visible tongue-flicks per span of time in seconds then converted to tongue-flicks per minute. For all other behaviors, only counts were recorded.

All behavioral variables were assessed in two temporal contexts: until first arm choice was made (“initial phase”) or until 4 min 45 sec had elapsed from the tegu’s emergence from the holding box at the base (“full phase”). The duration of full phase was determined by the tegu that had the shortest full-length video. Behaviors can vary significantly across the duration of an experimental test with reptiles, including phasic patterns and decay [62]. Further, during our initial tests of female tegus, individuals left the holding box and rapidly selected an arm (< 1 min) but then spent significant time re-investigating other areas of the maze. Therefore, segmenting analyses into phases provided a richer context for interpretation. Choice and choice penalty were only assessed during the initial phase of trials. All other variables were assessed in both the initial phase and full phase in each trial.

### Statistical analyses

Binomial tests were used for arm choice data. Two-way (sex, trial type) repeated measures ANOVA followed by pairwise comparisons (Student’s t tests) were used for rate of tongue-flicking, choice penalty score, and all behaviors for both trial phases. Because number of passes through each arm was scored, a separate set of analyses were conducted to assess if tegus differentially investigated the arms of the Y-maze using two-way (arm, trial type), repeated-measures ANOVA followed by pairwise comparisons. Statistical significance was set at P < 0.05, and marginal differences were also reported (0.05 < P < 0.1) given that sample size was modest but the experiment had a randomized repeated measures design.

## Results

### Y-maze performance

**Fig 1:**
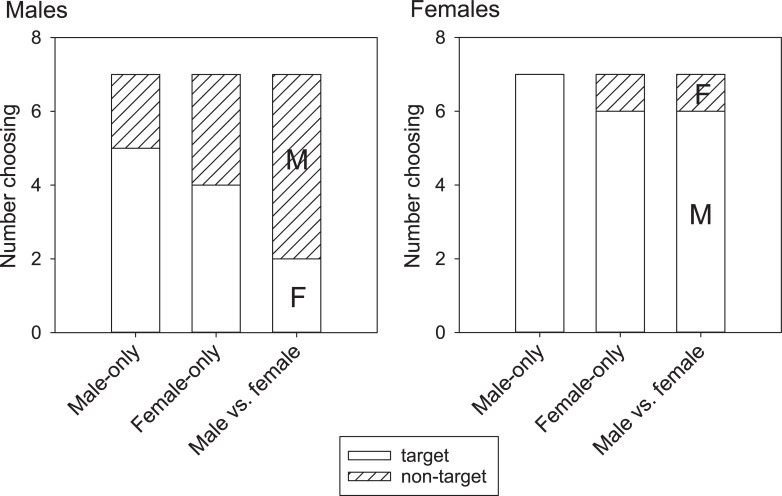
Choice results from Y-maze trials. In spring, females (left; *n* = 7) had a stronger preference for conspecific scent than did males (right; *n* = 7), especially when both male and female scent trails were present in the Y-maze. The only statistically significant choice was in the Male-only scent trials for females where 7/7 female tegus chose the male arm of the maze. The binomial probability for 6/7 successful choices is P = 0.054. In fall trials, male tegu performance in the Y-maze trials was similar to spring.

In spring trials, males (n = 7 for all trial types) did not show a preference for male scent trails (5/7, P = 0.224) or female scent trails (4/7, P = 0.5) (Fig 1). Further, males did not prefer either sex’s scent when presented with both simultaneously (2/7 chose female, P = 0.224). Females (*n* = 7 for all trial types) in spring showed a preference for male scent in the Male-only trials (7/7; P < 0.001) and marginal preferences in the Male vs. female trials (6/7 chose male scent; P = 0.054) and Female-only trials (6/7; P = 0.054) (Fig 1).

**Fig 2:**
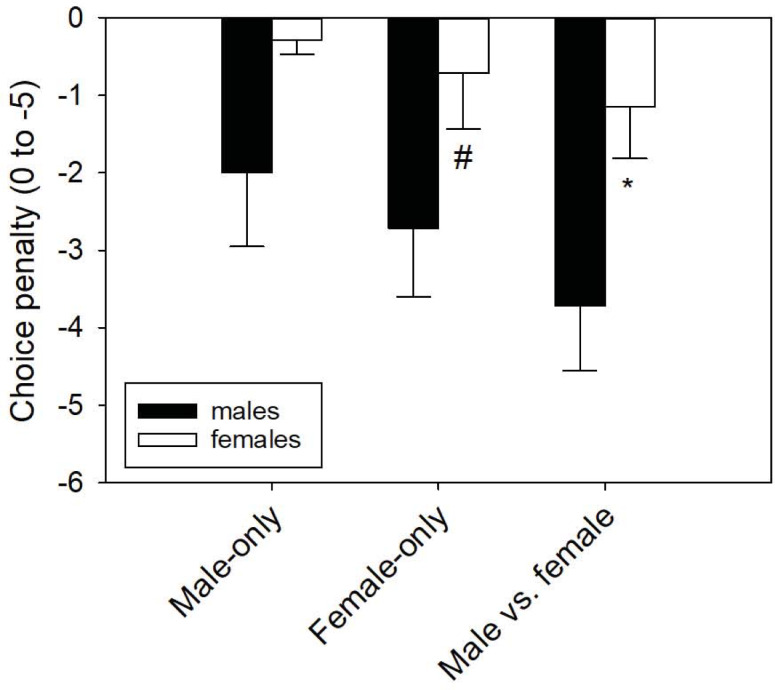
Choice penalty scores. Female tegus had less negative choice penalty scores compared to male tegus in the Y-maze. Choice penalty is a measure of how far the tegu moved in the non-target arm (unscented arm in Male- and Female-only trials; same-sex arm in Male vs. female). *P < 0.05 for comparisons within sex per trial type; #0.05 < P < 0.1. Bars are means (-SEM).

Choice penalty scores differed between the sexes (F_1,41_ = 16.17, P = 0.002)(Fig 2). Males had more negative choice penalty scores than females in the Male vs. female trials (q = 3.41, P = 0.021) and marginally lower scores in the Female-only trials (q = 2.65, P = 0.069) but not the Male-only trials (q = 2.27, P = 0.116).

**Fig 3:**
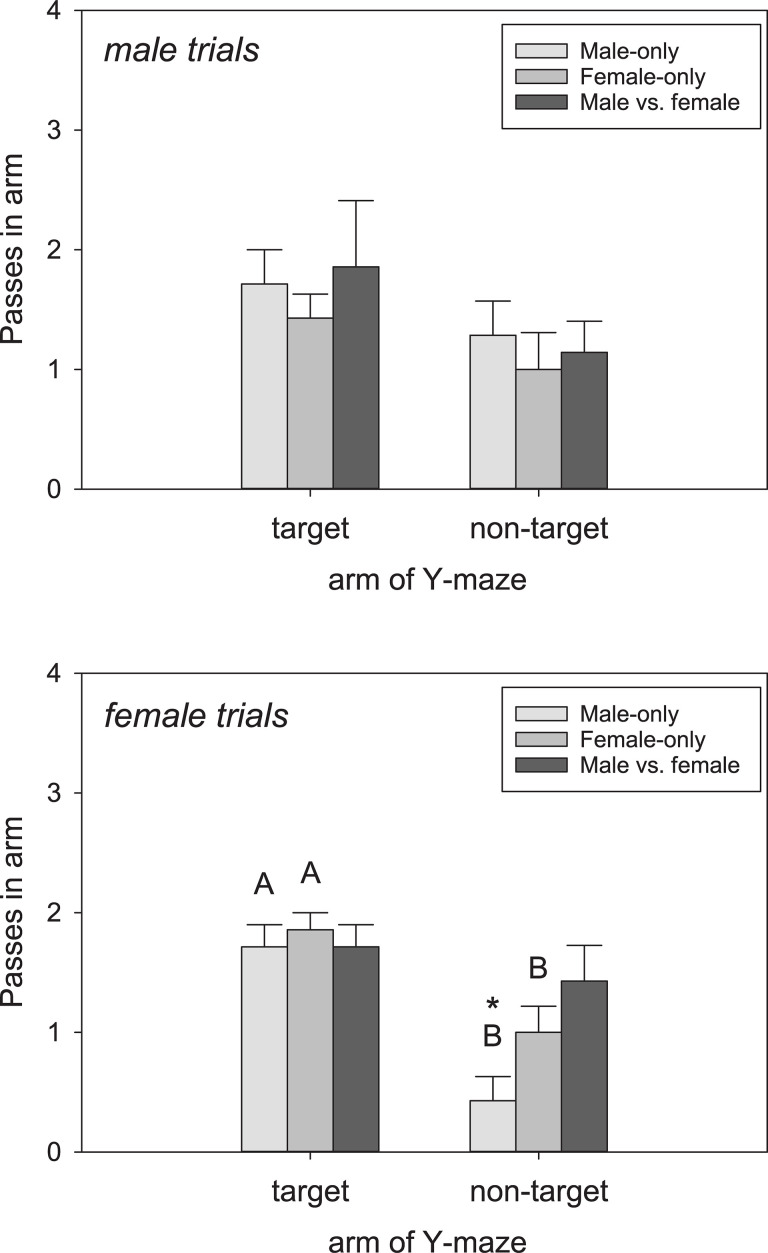
Number of passes through each arm of the Y-maze. Tegus differentially explored the Y-maze across the trial types based on number of arm passes. Top, males; bottom, females. Different letters (uppercase) represent statistically different (P < 0.05) pairwise comparisons between target and non-target arms within a trial type. Bars are means (+SEM).

**Fig 4:**
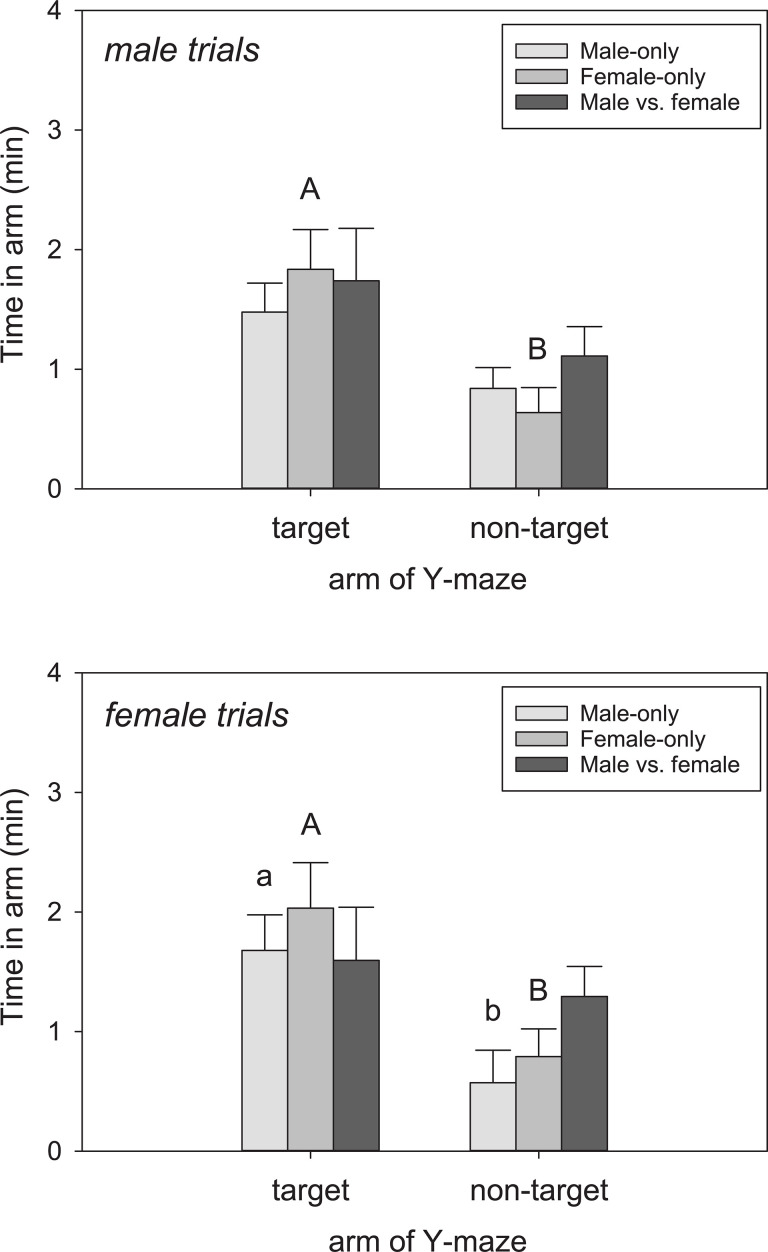
Time spent in each arm of the Y-maze. Tegus spent differential amounts of time in the target vs. non-target arms of the Y-maze across the trial types. Top, males; bottom, females. Different letters represent statistically different (P < 0.05) pairwise comparisons between target and non-target arms. Lowercase letters = 0.05 < P < 0.1. Bars are means (+SEM).

Considering the full phase trials, males did not pass through the target arm (= scented by conspecific) more often than the non-target arm across the trial types (F_1,41_ = 4.77, P = 0.072)(Fig 3). Males spent more time in the target arm than the non-target arm (F_1,41_ = 8.20, P = 0.029)(Fig 4), but only in the Female-only trials (q = 3.53, P = 0.022; Male-only, q = 1.88, P = 0.19; Male vs. female, q = 1.85, P = 0.20). Females in the full phase trials passed through the target arm more often than the non-target arm (F_1,41_ = 13.98, P = 0.010)(Fig 3), and there was a trial × arm interaction (F6,41 = 5.55, P = 0.02). Females passed more frequently through the target arm in the Male-only (q = 8.53, P < 0.001) and Female-only trials (q = 5.69, P < 0.001) but not the Male vs. female trials (q = 1.89, P = 0.19). Females also spent more time in the target arm (F_1,41_ = 53.47, P < 0.001)(Fig 4), specifically in the Female-only trials (q = 3.78, P = 0.018). There was a marginal difference in the Male-only trials (q = 2.75, P = 0.073) but not Male vs. female trials (q = 0.75, P = 0.64).

### Behaviors

**Fig 5:**
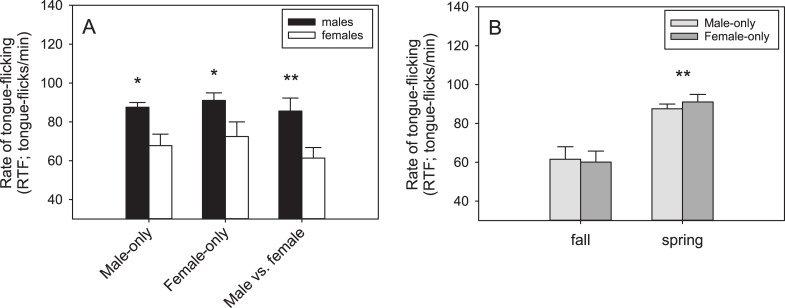
Rates of tongue-flicking by tegus in the Y-maze. A) Rates of tongue-flicking (RTF) in the initial phase of trials were higher in male tegus compared to female tegus. However, RTF was not different based on trial type within each sex. B) Males also have higher RTFs in spring than in fall during the initial phase. Bars represent means (+SEM). *P < 0.05, **P < 0.01.

**Fig 6:**
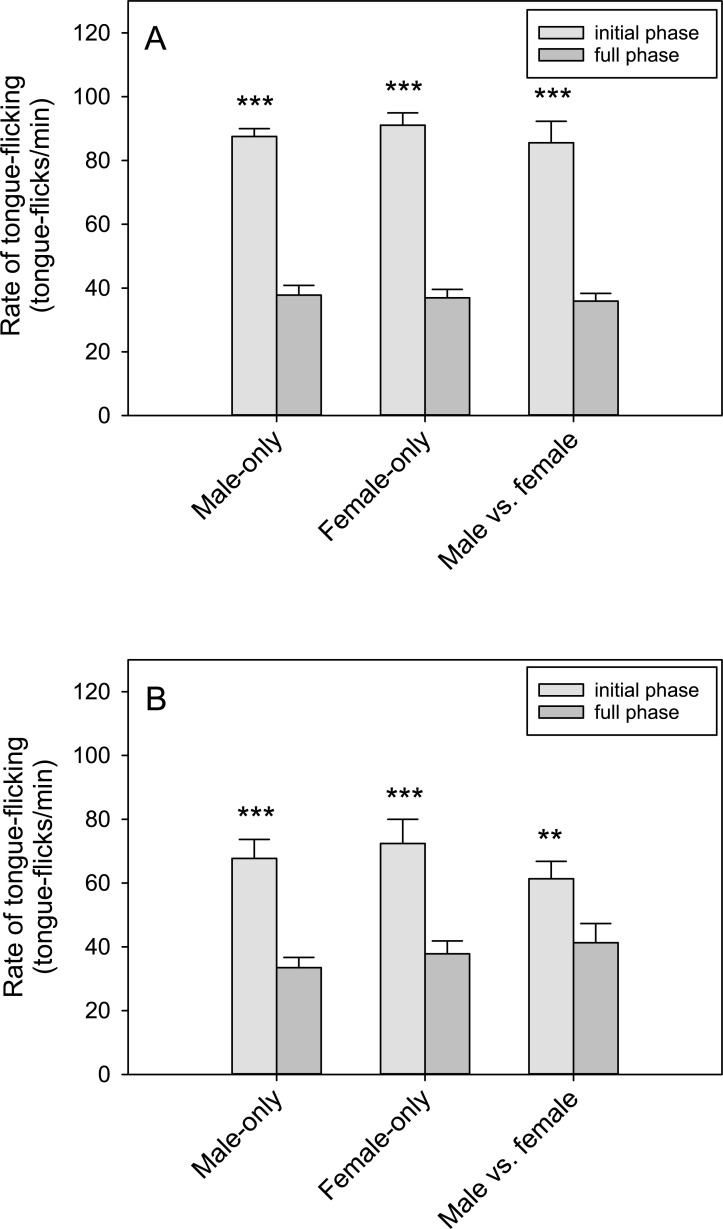
Phasic changes in rates of tongue-flicking by tegus while trailing. A) Male rates of tongue-flicking (RTF) were higher in the initial phase of trials than in the full phase across all trial types. B) Female RTFs showed the same pattern. Bars represent means (+SEM). **P < 0.01, ***P < 0.001.

In the initial phase of the spring trials, males exhibited a higher rate of tongue-flicking (RTF, tongue-flicks per min) compared to females across the trials (F_1,41_ = 22.11; P < 0.001)(Fig 5A). Trial type, however, had no effect on RTF (F_2,41_ = 1.06, P = 0.36). Male RTF was higher than female RTF in each trial type (Male-only, q = 3.53, P = 0.017; Female-only, q = 3.32, P = 0.024; Male vs. female, q = 4.31, P = 0.004). In the non-breeding season (fall), male RTF was lower than in spring (F_1,27_ = 26.24, P = 0.002) and in both trial types tested: Male-only and Female-only (q = 4.99, P = 0.004; q = 5.95, P = 0.001, respectively)(Fig 5B). Male vs. female trials were not conducted in fall. Tongue-flicking behavior was highly phasic. Males had higher RTFs in the initial phase compared to the full phase in every trial type (F_1,41_ = 138.74, P < 0.001; Male-only, q = 11.71, P < 0.001; Female-only, q = 12.74, P < 0.001; Male vs. female, q = 11.69, P < 0.001)(Fig 6A). The same was true for females (F_1,41_ = 81.66, P < 0.001; Male-only, q = 8.49, P < 0.001; Female-only, q = 8.58, P < 0.001; Male vs. female, q = 4.97, P = 0.003)(Fig 6B).

**Fig 7:**
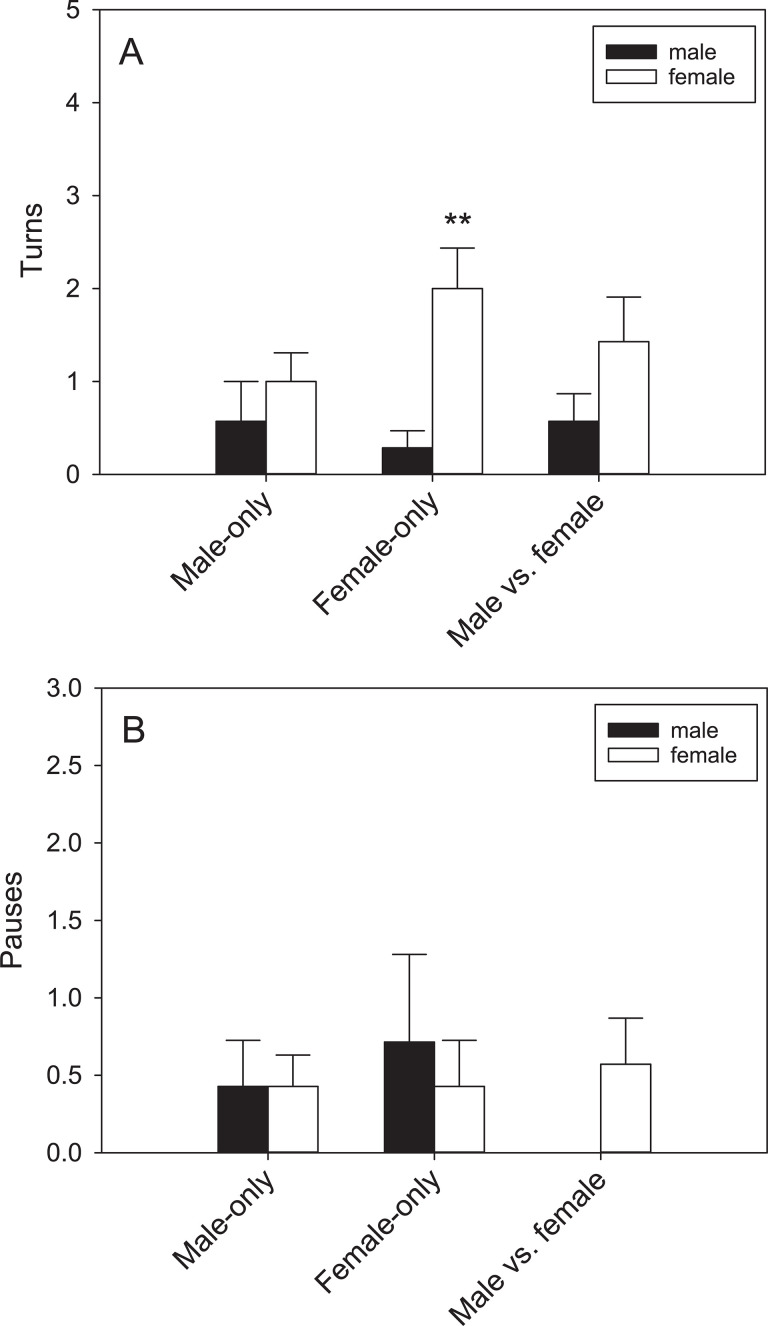
Turning behavior during scent trailing. A) Turning behavior was female-biased in tegus during the initial phase of trials, B) but pausing behavior was not. Bars represent means (+SEM). **P < 0.01.

**Fig 8:**
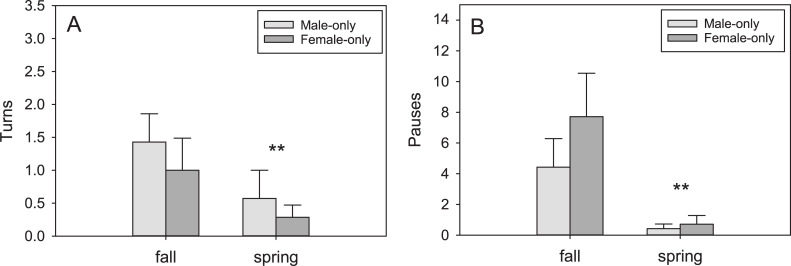
Seasonal differences in male tegu behaviors. Turning (A) and pausing (B) both decreased in frequency in the spring during the breeding season in the full-phase trials. Bars represent means (+SEM). **P < 0.01.

The tegus demonstrated quantifiable behaviors in the Y-maze trials in the presence of conspecific scent trails, but differences based on sex or trial type were only detected in the initial phase. Turning behavior was female-biased (F_1,41_ = 27.19, P < 0.001), with females turning more frequently than males but only in the Female-only trials (q = 5.61, P < 0.001)(Fig 7). Males showed seasonal variation in turning and pausing behavior in the full phase trials. Males turned more often in fall than spring (F_1,27_ = 7.56, P = 0.033)(Fig 8A). Males also paused more frequently in fall (F_1,27_ = 6.75, P = 0.041)(Fig 8B). No other differences were significant for any of the behaviors scored.

## Discussion

Argentine black and white tegus use conspecific scent during the breeding season, and their responses to conspecific odor are sex-specific, including some chemosensory behaviors. Our findings suggest that conspecific odor drives trailing behavior in this invasive species during its breeding season in Florida (March-May). Both sexes spent disproportionate amounts of time investigating conspecific scent compared to areas lacking scent. Females in sum appear to have a high degree of precision while sampling conspecific scent trails. Compared to males, female behavior may be more driven by conspecific chemical cues: they explored the maze less (less negative choice penalty scores), spent more time and effort (arm passes) with target scent, and employed behaviors that may facilitate this ability (increased pausing and turning). Males, however, appear to employ broader searching strategies than females (e.g., more exploration of the entire environment, greater RTFs) and may not preferentially search for female scent trails. We also detected significant, phasic changes in rates of tongue-flicking (RTF) as has been demonstrated in other reptiles engaged in chemosensory searching [61,62], and we documented two behaviors (pausing, turning) consistent with similar studies where reptiles employ specific repertoirs of behaviors to facilitate searching [60, 62].

Our study has two shortcomings. First, our sample size was modest, and therefore our inferences are limited in scope. However, we believe that our findings are significant in their novelty and are bolstered by the fully randomized, repeated-measures design we implemented. Second, our tegus had been in outdoor enclosures for various spans of time that may have affected individual responses. We could not adequately control for this other than the study design we mentioned above. We added study animals to our sample size piecemeal until we could conduct the planned studies in the same year to control for year-to-year variation. Given that novel findings in a given species or system establish precedence for future experimentation, it is important to acknowledge these aspects of our study.

Lizards are well-documented to rely on multimodal signals in mate choice, and sexual behaviors can be significantly altered by presenting signals in combination versus isolation [e.g., 63]. There are also evolutionary patterns in lizards suggesting that as sexual dimorphisms in visual signals (e.g., coloration) were lost, a stronger reliance on and response to conspecific chemical cues evolved [e.g., 64,65]. In the management of invasive or pest insect species, pheromones often interact with other signals to affect conspecific behavior and alter trap efficiency [e.g., 66,67]. As such, an exploration of whether coupling conspecific scent with a source of visual signals (e.g., static models) is warranted and could lead to greater rates of sex-specific removal of *S*. *merianae* during the breeding season. We documented classic chemosensory behaviors in tegus in this study (phasic RTF, searching behaviors) when only chemical cues from conspecies were available in the environment. If such behaviors increase in intensity when multiple conspecific signals are available to focal animals, more effective tools could be designed to aid in management.

Overall, female tegus demonstrated stronger trailing behavior by following both male and female scent trails and exhibiting greater decisiveness than males in arm choice. In general, male reptile behaviors directed toward conspecifics are sex-specific and have been documented in many squamate species in natural and experimental settings [9, 15, 60, 68]. These male-biased findings are informative to both basic and applied research. But, the potential implications of our findings in females are considerable because in most vertebrate populations, female reproduction is the ultimate driver of state variables such as population size [69], and removing females is often a principle goal for wildlife managers of invasive animal species. Centering management efforts on targeting and reducing the number of females could hasten long-term population reduction or eradication. Across a broad range of invasive and noxious insects, sex-specific behavior elicited by pheromones has been leveraged to improve control methods and consequently increase the probability of meeting management objectives [26]. Application of species-specific chemical signals to reduce impacts of invasive vertebrates has been less common but merits further attention. Johnson et al. [70] found that a synthesized pheromone induced upstream movement in female sea lampreys (*Petromyzon marinus*), thereby increasing capture efficiency at upstream trapping sites. Similarly, Takács et al. [28] found sex hormones functioned as sex attractant pheromones in house mice (*Mus musculus*) and brown rats (*Rattus norvegicus*), and these attractants increased capture efficiency. Manipulation of pheromone levels in invasive snake species, such as brown treesnakes (*Boiga irregularis*), has been explored and shows promise for management [71].

Life history parameters of an invasive species are major considerations in designing effective management approaches [72]. Which tools and when they are deployed can have varying degrees of success in seasonally mating species (e.g., round gobies, *Neogobius melanostomus*)[73]). This is especially true when evaluating tools that mimic or manipulate mating signals where significant interactions can exist between a tool (e.g., pheromone lures) and a given season (e.g., stink bugs, *Halyomorpha halys*, [74]; lampreys, *Petromyzon marinus*, [75]). Seasonality of trapping strategies is an often overlooked element in the management of terrestrial invasive vertebrates, particularly amphibians and reptiles. When considering the use of scent in management of squamates and possible seasonal constraints, studies in common garter snakes (*Thamnophis sirtalis*) are illuminating [15]. The sensory system required to detect and respond to sex pheromones in squamates changes dynamically due to seasonal influences, primarily temperature [76,77]. Most importantly, male *T*. *sirtalis* only respond to female sex pheromones during the breeding season because of specific upregulation of this pheromone detection system [78,79]. Our results suggest that male Argentine black and white tegus are more responsive to conspecific scent during the breeding season (March-May) than the non-breeding season (September-November). Male tegus had lower rates of tongue-flicking (RTF) and increased exploratory behaviors (turns, pauses) in the non-breeding season. In squamates, RTF is a proxy for chemosensory sampling rate and is used diagnostically to determine general levels of interest in and responsiveness to a specific type of chemical cue. If tegus are less interested in (or responsive to) conspecific odor in the non-breeding season, reproductive chemical cues could have limited utility outside the window of time in which this (or any) invasive reptile species is searching for mates.

For *S*. *merianae* and other invasive terrestrial reptiles where trapping is the prevailing management tool [33, 80], trapping efficiency for females could be increased by developing chemosensory lures used in concert with trapping efforts in the breeding season. An important step toward this goal would be to develop a pheromone lure capable of being readily tested under field and laboratory settings. Extraction of lipids from shed skins isolates relevant conspecific signals and solubilizes them in a dispersible solvent, hexane [81], and this could provide a medium usable for Y-maze or pen-based trap preference trials. If either of these low cost experiments provide confirmatory results, testing under more resource-intensive, large-scale field conditions could be pursued. Other factors, however, such as lability of chemical lure trails, would need to be assessed.

Mainland eradications of invasive reptiles are rare due to myriad factors [82–84], and to our knowledge zero successful eradications of an established invasive reptile population have occurred in such areas. Low detection probabilities make it difficult for researchers to assess effectiveness of population management efforts thereby discouraging evaluations of new candidate methods [85]. Leveraging the chemical ecology of an invasive species via pheromone manipulation may be most effective for insular efforts when employed as part of an Early Detection and Rapid Response (EDRR) program. Maximizing trapping efficiency in these scenarios could be the deciding factor between eradication and population establishment [85].

## Supporting information

S1 Fig Black and white Argentine tegu (*Salvator merianae*) inside the experimental Y-maze apparatus. The tegu is located at the terminus of the initial passageway (base) of the maze. The placard indicates the tegu’s unique number and sex. All trials were conducted at the U.S. Department of Agriculture, National Wildlife Research Center, Florida Field Station, Gainesville, Florida, USA.
(DOCX)

S2 Fig (JPG)

S1 Table All data collected from the behavioral trials. In this file, all of the behavioral data obtained from the video recordings are available. The tabs in the sheet organize the data by season, and the last tab defines the behavioral and other variables recorded in each trial.
(XLSX)
