# Supplementary material for: Conspecific chemical cues facilitate mate trailing by invasive Argentine black and white tegus
Source: PLoS One. 2020 Aug 12;15(8):e0236660. doi: 10.1371/journal.pone.0236660 (PMC7423067; doi:10.1371/journal.pone.0236660)

**Fig S1.** **Black and white Argentine tegu (*Salvator merianae*) inside the experimental Y-maze apparatus.** The tegu is located at the terminus of the initial passageway (base) of the maze. The placard indicates the tegu’s unique number and sex. All trials were conducted at the U.S. Department of Agriculture, National Wildlife Research Center, Florida Field Station, Gainesville, Florida, USA.


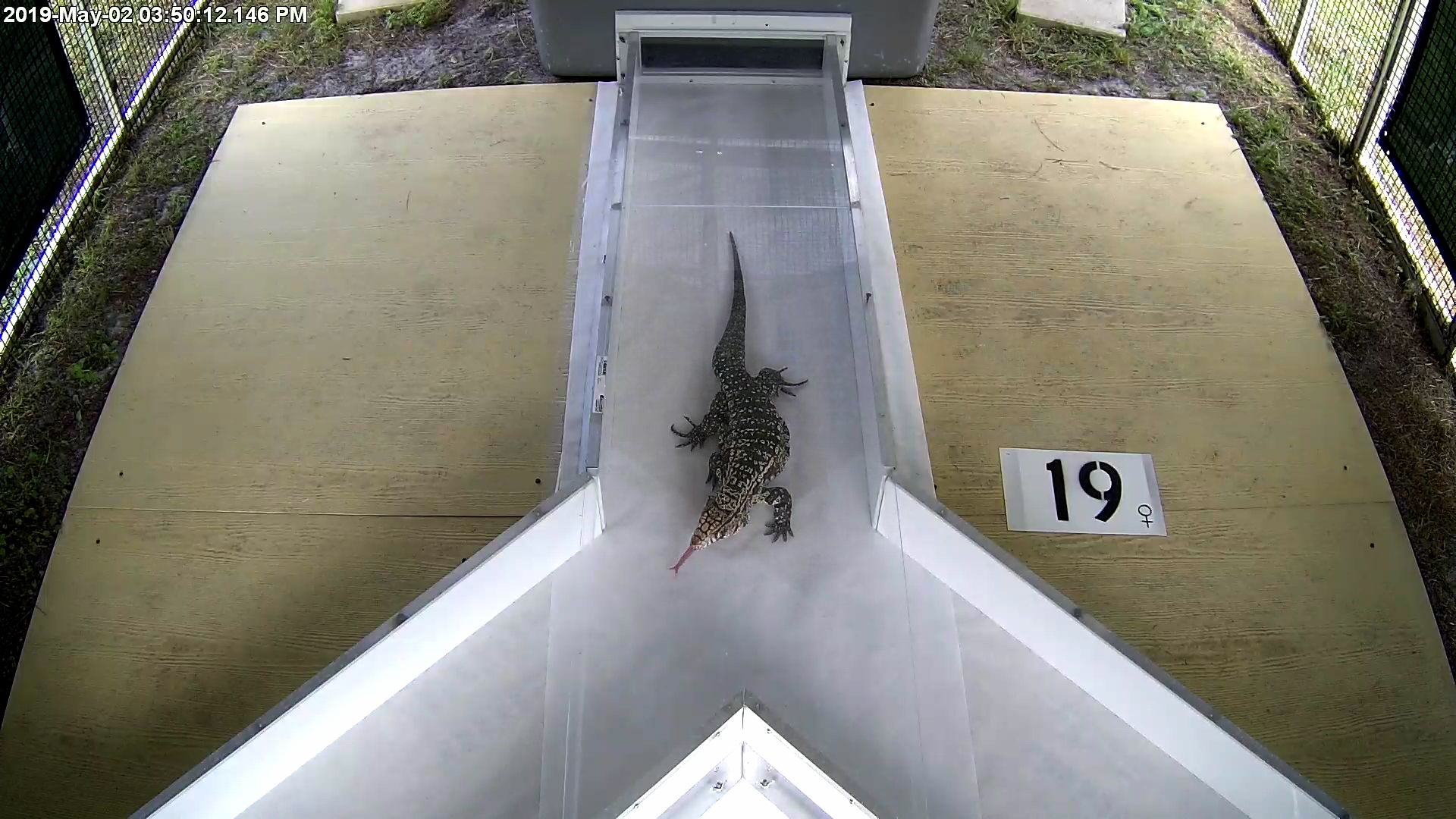

Supplement: S1 Fig — The tegu is located at the terminus of the initial passageway (base) of the maze. The placard indicates the tegu’s unique number and sex. All trials were conducted at the U.S. Department of Agriculture, National Wildlife Research Center, Florida Field Station, Gainesville, Florida, USA. (DOCX) [file pone.0236660.s001.docx]
