# Supplementary figures and images for: Conspecific chemical cues facilitate mate trailing by invasive Argentine black and white tegus
Source: PLoS One. 2020 Aug 12;15(8):e0236660. doi: 10.1371/journal.pone.0236660 (PMC7423067; doi:10.1371/journal.pone.0236660)

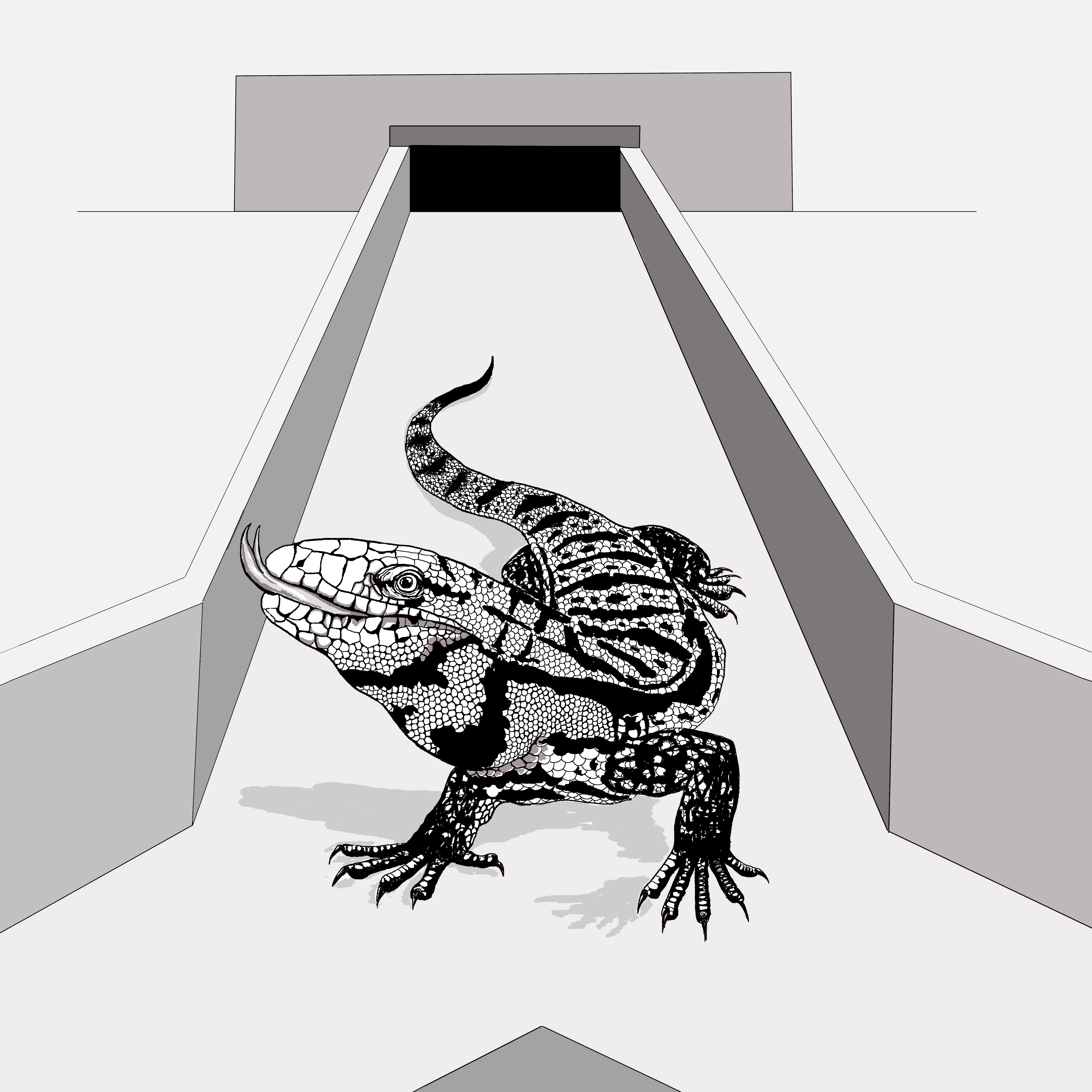

Supplement: S2 Fig — (JPG) [file pone.0236660.s002.jpg]
